# Droplet motion on sonically excited hydrophobic meshes

**DOI:** 10.1038/s41598-022-10697-9

**Published:** 2022-04-26

**Authors:** Abba Abdulhamid Abubakar, Bekir Sami Yilbas, Hussain Al-Qahtani, Ammar Alzaydi

**Affiliations:** 1 Mechanical Engineering Department, King Fahd University of Petroleum and Minerals, Dhahran, 31261 Saudi Arabia; 2 Interdisciplinary Research Center for Renewable Energy & Power Systems, KFUPM, Dhahran, 31261 Saudi Arabia; 3 K.A. CARE Energy Research & Innovation Center at Dhahran, Dhahran, Saudi Arabia; 4 Turkish Japanese University of Science and Technology, Istanbul, Turkey

**Keywords:** Engineering, Mechanical engineering

## Abstract

The sonic excitation of the liquid droplet on a hydrophobic mesh surface gives rise to a different oscillation behavior than that of the flat hydrophobic surface having the same contact angle. To assess the droplet oscillatory behavior over the hydrophobic mesh, the droplet motion is examined under the external sonic excitations for various mesh screen aperture ratios. An experiment is carried out and the droplet motion is recorded by a high-speed facility. The findings revealed that increasing sonic excitation frequencies enhance the droplet maximum displacement in vertical and horizontal planes; however, the vertical displacements remain larger than those of the horizontal displacements. The resonance frequency measured agrees well with the predictions and the excitation frequency at 105 Hz results in a droplet oscillation mode (n) of 4. The maximum displacement of the droplet surface remains larger for the flat hydrophobic surface than that of the mesh surface with the same contact angle. In addition, the damping factor is considerably influenced by the sonic excitation frequencies; hence, increasing sonic frequency enhances the damping factor, which becomes more apparent for the large mesh screen aperture ratios. The small-amplitude surface tension waves create ripples on the droplet surface.

## Introduction

Liquid droplets and their behavior on surfaces are of interest in various applications such as biomedicine^1^, combustion^2^, and self-cleaning^3^. In general, the balance among the external, surface tension, gravity, and viscous forces can govern liquid droplet motion on the surfaces. Force due to surface tension is related to the stiffness of the dropet^4^ while viscous force is associated with the damping of the motion under viscous dissipation^5^. The gravitational force causes droplet puddle, which is related to the droplet size and the contact angle, provided that the puddling becomes negligible for the droplets having sizes comparable or less than the capillary length^6^. The external force created by an acoustic excitation gives rise to a droplet wobbling while creating a fluid motion^7^. Hence, the sessile droplet behavior can be manipulated by the acoustic waves, which causes droplet oscillatory motion. The acoustic power level of the excitation, surface tension, and viscosity of the droplet fluid remain important for the lateral and vertical motions of the droplet on the hydrophobic plane. The harmonic, subharmonic, and super harmonic mods of oscillations can occur at sonic excitation frequencies within the range of the Rayleigh-Lamb inertia-capillary frequencies^8^; however, for low-frequency oscillations, the droplet behavior is mainly governed by the droplet size and the tension between the fluid–solid interface^9^. Moreover, the droplets demonstrate different behavior on the oscillating liquid films and a permanent bouncing can result over the oscillating liquid film. Based on the excitation frequency and the amplitude of the excitation, mainly the Rayleigh frequencies influence the droplet bouncing mechanism^10^. The nonlinear hydrodynamic effects can be created in the droplet fluid by the Rayleigh surface acoustic waves and the assumption of Stokes model for fluid motion fails to predict the fluid velocities at high oscillation frequencies because of the nonlinear hydrodynamic effects created inside the droplet fluid^11^. Hence, rocking, and ratcheting of droplets can occur over the surface depending on the non-linear hydrostatic effect created within the droplet. The vertical and horizontal motions of the droplet motion are limited with the droplet pinning forces due to the contact angle hysteresis and interfacial resistance. This situation is usually observed at low amplitude and frequency oscillations. However, as the excitation frequency increases the droplets can overcome the pinning forces and move into a ratcheting mode. Furthermore, as the excitation frequency increases further, the droplet can jump and break into newborn droplets while ejected from the surface^12^. As the surface wetting changes to a superhydrophobic state, then, the sonic excitation can cause jumping of the droplets at a ratcheting mode. The impacting droplet on the oscillating surface undergoes a complicated behavior such that increasing Weber number significantly affects the retraction forces and the droplet rebound height can be attenuated because of the instability of the droplet rim^13^. However, for the hydrophilic surfaces, the droplet behavior differs considerably under the sonic excitation. The droplet remains pinned on the surface and it shows rather sliding and climbing behavior than the rebounding; hence, depending on the sonic frequencies, the droplet shape change and the climbing height varies considerably^14^.

The hydrodynamic interference of droplets with the external sonic excitation can create a jetting effect on the droplet motion. The combination of increasing surface hydrophobicity, reducing droplet fluid viscosity, and increasing fluid surface tension enhances the velocity of the droplet during the jetting motion^15^. The droplet jetting behavior can be modified as the droplet motion is arranged over the thin liquid film, which is excited by the sonic waves. The thin film oscillation under the surface (acoustic) waves can result in an irregular droplet wobbling behavior, which causes the complicated acousto-fluidics-phenomena while creating internal streaming in the droplet fluid and changing the droplet jetting behavior^16^. In some cases, the droplet can adhere under the influence of periodic oscillations, and droplet motion becomes droplet size and oscillation frequency dependant. The transversal motion can occur predominantly within the range of the droplet's first natural frequency at which the asymmetric shape deformations are encountered. However, as the frequency increases, the adhering droplet breaks into smaller droplets^17^. From the application point of view, the sonic excitation of droplets over surfaces can find solutions for some engineering problems such as surface cleaning and corrosion prevention of electronic devices^18^ and microfluidics of droplet displacement in micromachining processes^19^. In such practical applications, the flow field developed within and around the sonically excited droplets remains critical for droplet mobility over the surfaces. Since the precise experimental investigation for internal fluidity and external flow around the excited droplets is extremely challenging, the numerical predictions of the flow field remain fruitful^20^. Nevertheless, accurate flow predictions can shed light on the droplet behavior over the vibrating surfaces and provides useful guidance for experimental investigations.

The sonic excitation of a droplet over hydrophobic surfaces can give rise to alteration of interfacial contact and resistance that influences the pinning force over the surface. The creation and evolution of surface tension and gravity waves and hydrostatic pressure variation in the droplet fluid modify droplet shape dynamically during the acoustic excitations. Depending on the acoustic excitation frequencies and amplitudes, the droplet can undergo various modes of oscillations. In some modes, droplet jumping and breaking into newborn small droplets become unavoidable. Although droplet behavior on hydrophilic and hydrophobic surfaces under acoustic excitations has been studied^6,21–23^, the sonic wave direct interaction with a droplet fluid and resulting droplet motion on hydrophobic mesh surfaces were left for future study. As a liquid droplet is dispensed over the mesh surface, the interfacial area of the droplet over the mesh reduces significantly because of the open screen area of the meshes. The droplet fluid remains exposed to surrounding air over the open screen of the meshes and the sonic excitations from the droplet bottom result in pressure waves directly imposed to the droplet exposed surfaces. This alters the droplet behavior from those exposed to the acoustic excitations, such as Lamb waves, over the hydrophobic surfaces. Consequently, investigation of the dynamic response of the droplet under direct sonic excitations becomes necessary to evaluate the modes of droplet oscillation, the resonance frequency, and the time evolution of the maximum height. Hence, the droplet behavior over the hydrophobic mesh surface is studied and the influence of the sonic excitation frequencies on the droplet characteristics is examined. The metallic meshes with different screen areas are hydrophobized and a high-speed recording facility is utilized to evaluate the droplet response to the sonic excitations.

## Experimental

**Figure 1:**
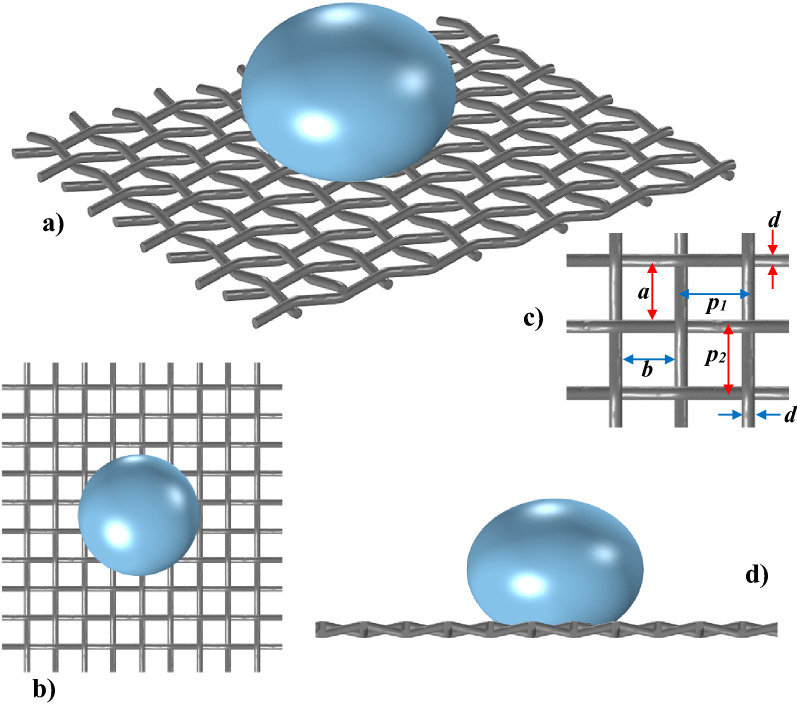
View of a sessile drop on hydrophobized mesh: (**a**) 3D view, (**b**) top view, (**c**) mesh dimensions, and (**d**) side view.

**Figure 2:**
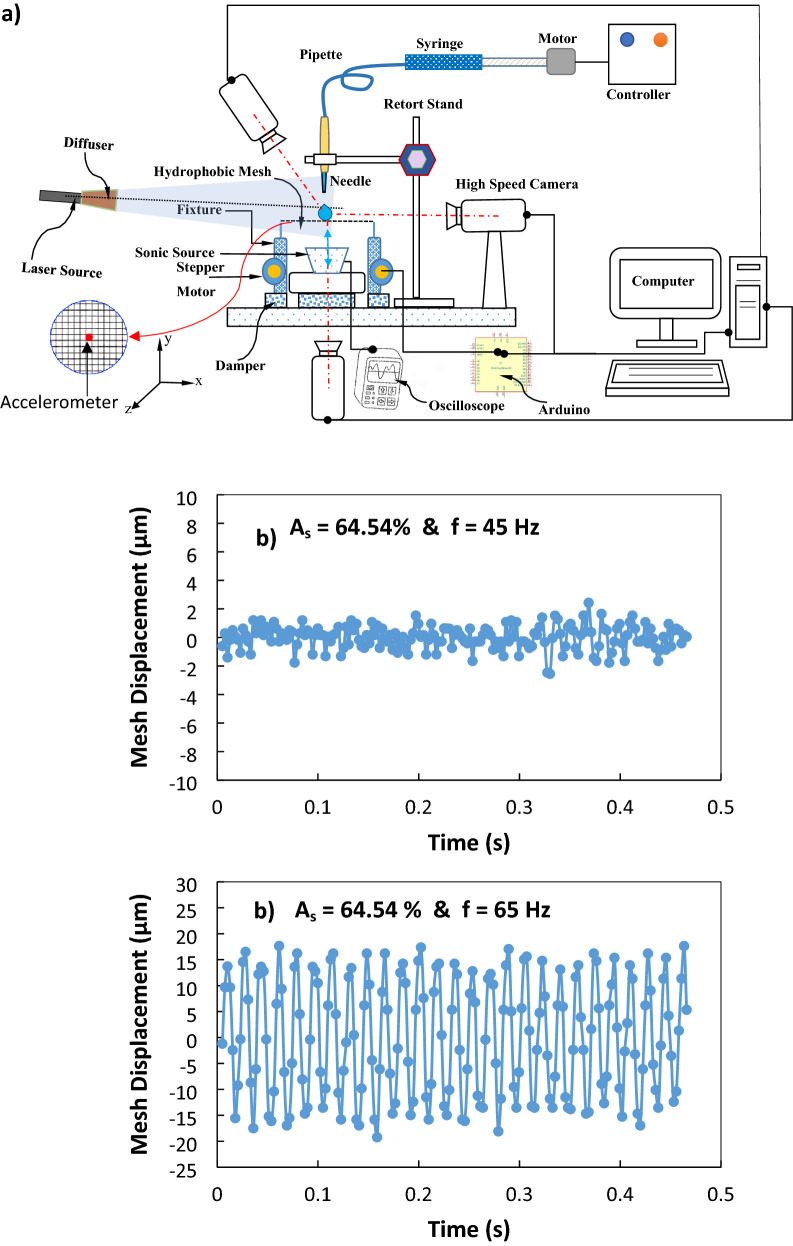
(**a**) Schematic view of the experimental setup. (**b**, **c**) Accelerometer readings for the vertical displacement of mesh screen structure with A_s_ = 65.54% and frequency of: (**a**) 45 Hz and (**b**) 65 Hz.

The metallic meshes formed from Steel (AISI 304) wires having 250–420 μm diameter were used in the experiments. Figure 1a–d show the schematic presentation of the water droplet over the hydrophobized mesh. The mesh has square screen spacings and the width size (a) changes for different mesh screen configurations; hence, different sizes of the screens were used in the experiments, which include the open aperture ratio of 61.17%, 62.34%, and 64. 51%. The mesh screen open aperture ratio was defined through the mesh open screen area ($a\times b$, where *a* = *b*) and the pitch area (${p}_{1}\times {p}_{2}$, where p_1_ = p_2_), ${A}_{s}=\frac{a\times b}{{p}_{1}\times {p}_{2}}\times 100\mathrm{\%}$ (Fig. 1c). The edges of the mesh screen are fixed delicately around a circular solid ring using metallic glue and no mechanical stretching is applied to the meshes during the experimental tests. The ring and mesh assembly was a part of the fixture which was tightly fixed on the test table with the vibration isolation foams.The functionalized silica particles were used to hydrophobize the mesh wire surfaces. The solution was prepared with a mixture of 14.4 mL of ethanol, 1 mL of de-ionized water, and 25 mL of ammonium hydroxide, and later 3:4 molar ratio of silane was added^24^. The solution was placed in coating equipment (Biolin Scientific) and the samples were coated, and later the coated samples were placed in the laboratory fume hood for 2 h allowing volatile compounds to evaporate. The coated surface was assessed using the scanning electron microscope (SEM, JEOL 6460) and atomic force microscope (AFM, Flex-Axiom, Nanosurf). The wetting characteristics of the coated mesh surfaces were evaluated by using a Goniometer (Kyowa, model DM 501). To create sonic excitations under the mesh, a fixture was designed and manufactured. Figure 2a shows a schematic view of the experiments. The loudspeaker (Edifier Inc.), which was operated at 9 V (DC) and 0.44 A, was used to create the adjustable sonic excitation frequencies under the hydrophobized mesh. The accelerometer (KX132-1211 accelerometer, Kionix) was used to ensure the frequency and amplitude of the excitation sonic waves. The accelerometer is placed at the center of the mesh structure which exactly coincides with the location of sessile droplets to calibrate the mesh displacement during the sonic excitation. However, the accelerometer is placed on the mesh structure in the absence of the droplet. This is because, the experimental data shows that the droplet weight is significantly smaller than that of the mesh structure. The mesh displacements with time for two excitation frequencies are shown in Fig. 2b and c for the screen arrangement of A_s_ = 65.54%, respectively. Almost regular oscillatory behavior is observed for the mesh response to external excitation. However, increasing excitation frequency increases substantially the oscillation amplitude, which is expected to influence the droplet impact behavior particularly at higher frequencies. Similarly, several tests were conducted initially to assess the droplet response to the excitation frequencies and powers. The excitation frequencies within the range of 40 Hz to 150 Hz were selected. The selection was based on the magnitude and frequencies of the oscillations created on the droplet. It is worth mentioning that by lowering the excitation frequency beyond 40 Hz, the droplet did not demonstrate notable oscillations, and the droplet mitigation from the hydrophobic mesh either by rolling or expelling (jumping) occurred as the excitation frequency was increased beyond 150 Hz. A high-speed camera system was used recording the droplet behavior during the sonic excitations. The camera was operated at 5000 fps having a resolution of 1280 × 800 pixels with a pixel size of 14 µm × 14 µm. The tracker program was utilized to extract and analyze the recorded data. Uncertainty analysis was carried out evaluating the experimental uncertainty. The tracker program enabled to extract the high-speed camera data in terms of droplet lobe displacements and periods of displacement oscillations. It is worth mentioning that the experiments were repeated 12 times to ascertain the 96% confidence level and the experimental uncertainty is evaluated accordingly. The variation of the droplet lope size at each data point is evaluated from the tracker program. The discrete data variation at each time response of droplet lope during the experimental repeats is considered as a discrete random variable, which is arranged in a series to obtain the probability function. The Gaussian function is considered to resemble the probability function. In general, the uncertainty can be written as: ${\sigma }_{u}=\sqrt{{\int }_{{x}_{o}}^{{x}_{ne}}{\left(x-{x}_{e}\right)}^{2}f\left(x\right)dx}$, 25 where, x is the variable obtained from the data set, ${x}_{e}$ is the mean value of the variable, ne being the number of data points in the data set, and $f\left(x\right)$ is the probability function (Gaussian form). The probability distribution was used to determine the function diameter. The bias error was estimated as 0.5 pixels because of the small peaks in the distribution spectrum, which were difficult to evaluate with accuracy. The uncertainty was estimated in the order of 3.5%.

## Results and discussion

A droplet behavior on a hydrophobic mesh, which is externally excited by sonic waves, is investigated. The metallic meshes are hydrophobized by depositing functionalized nano-silica particles. The influence of the sonic excitation frequency and mesh screen open area on the droplet motion in terms of natural and resonant frequencies, mode of oscillation, and droplet maximum displacement are analyzed.

### Surface characteristics of meshes

**Figure 3:**
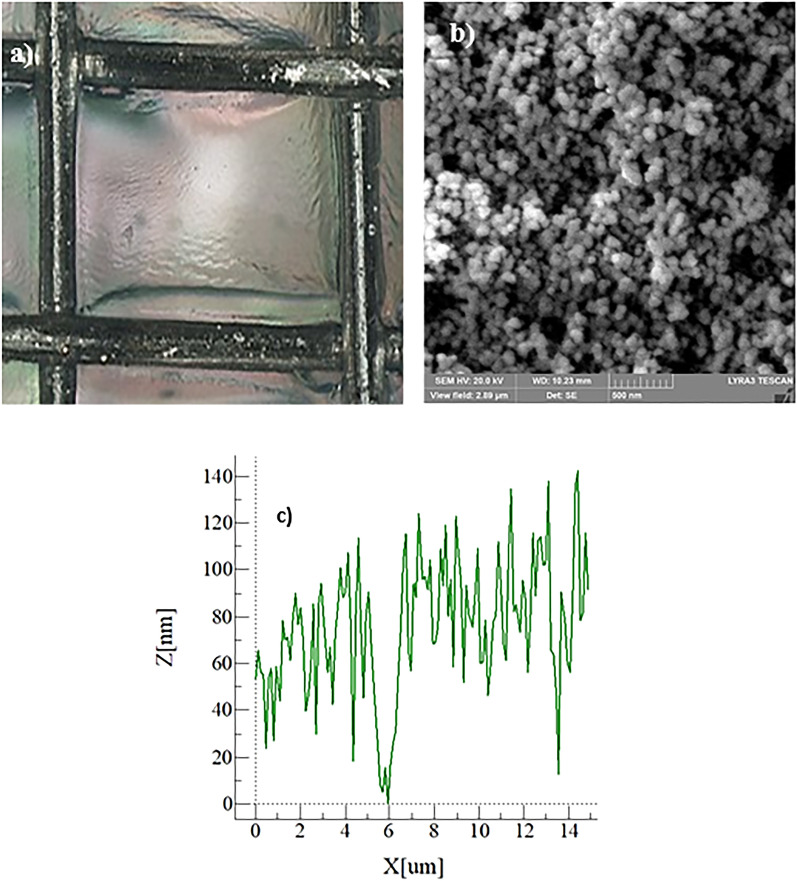
Optical and SEM illustrations and AFM line scan of mesh: (**a**) optical image of the mesh after coating, (**b**) SEM micrograph of coated mesh wire, and (**c**) AFM line scan of coated mesh wire.

**Figure 4:**
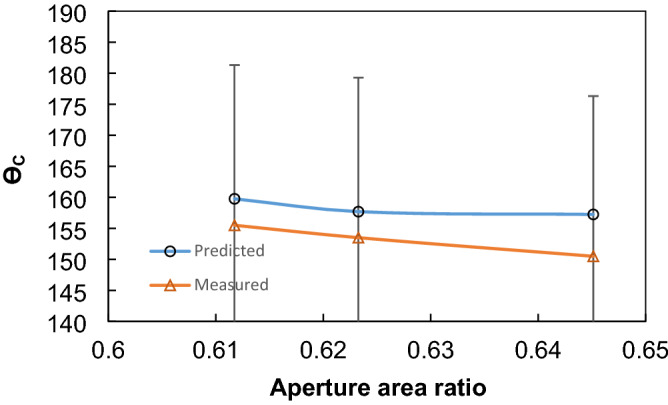
Droplet apparent contact angle predicted and obtained from the experiments with mesh screen open aperture ratio (*A*_*s*_).

The optical image of the hydrophobized metallic mesh is shown in Fig. 3a while the SEM micro-image of the coated surface is shown in Fig. 3b. The line scan of the surface obtained from the atomic force microscope is also shown in Fig. 3c. The meshes consist of uniformly extended square open screens and hydrophobized wires (Fig. 3a). The hydrophobized wire surface composes of agglomerated nano-silica particles forming nanosized webs-like structures (Fig. 3b). Since the particle agglomeration appears to be non-uniform over the surface, it creates nanosized cavities and pillars like topology forming the texture. This can be seen from Fig. 3c (surface line scan). The presence of nano-peaks and nano-valleys are the result of the agglomeration of the nano-particles during deposition. The maximum peak height is about 140 nm and the average surface roughness is 94 nm while the roughness parameter is almost 0.52. It should be noted that the roughness parameter is the same as the ratio of pillar area over the projected surface area. The roughness parameter partly resembles the texture morphology of the surface and as the roughness parameter approaches unity, the surface becomes highly rough, and roughness parameter zero yields no-pillars over the surface, i.e. perfectly smooth surface. The contact angle on the mesh surface is measured by a Goniometer. However, establishing the droplet contact line on the surface can differ from that of the smooth hydrophobic surface. This is because the droplet partially inflects into the mesh through the mesh screens. The apparent contact angle is considered for the measurements, which can change with the mesh size^25^. After considering the mesh geometry composes of isotropic structures and the fluid inflection in the mesh screens is small, then the apparent contact angle can be written as^26^:

1 $$\mathrm{cos}{\theta }_{app}=\frac{1}{{D}_{n}}\left(\pi -{\theta }_{c}\right)\mathrm{cos}{\theta }_{c}+\frac{1}{{D}_{n}}\mathrm{sin}{\theta }_{c}-1$$

where ${D}_{n}=\frac{(d+{w}_{avg})}{d}$ is the pitch length of the mesh normalized by the wire diameter (*d*), *w*_*avg*_ represents the aperture width of the mesh screen open area (spacing of two adjacent warp/weft—wrap is the wires run lengthwise of the mesh and weft is that wires run across the mesh—corresponding to the center-plane of the open mesh screen), and ${\theta }_{c}$ is the contact angle over the mesh material (steel). Figure 4 depicts the contact angle predicted and measured with open screening area ratios. It is worth mentioning that the open screen aperture ratio represents the ratio of aperture area to the pitch area, i.e. ${A}_{s}=\frac{a\times b}{{p}_{1}\times {p}_{2}}\times 100\%$, here *a* and *b* are the horizontal and vertical gaps in the mesh screen while *p*_*1*_ and *p*_*2*_ are the gaps of the centroid of two adjacent mesh wires in horizontal and vertical directions (Fig. 1c)^27^. The deviation between the predictions and the result of Eq. (1) occurs and it is associated with the considerations incorporated in formulating Eq. (1), i.e. mesh sizes are not precisely the same and they are not exactly distributed uniformly over the entire mesh. In addition, droplet fluid inflection in the mesh screen open area may not be small for the meshes used in the experiments. The mesh size and the apparent contact angle measured for different mesh screen open areas are provided in Table 1. Increasing the screen aperture ratio incorporated in the current study lowers the contact angle. The contact angles are measured by adopting the precision measurement method^28^. In this case, the circumference of the droplet image obtained from the Goniometer is fitted in a mathematical function. The horizontal line is introduced to account for the contact line of the droplet. In some cases, the horizontal line may not exactly correspond to the droplet contact line; hence, a correction angle (${\alpha }_{BL}$) is introduced to fix the exact contact line over the surface. The droplet contact angle is evaluated from the function fitted via:

**Table 1:** Specification for wire mesh size adopted. a and b are horizontal and vertical wire spacings in a single mesh cell, and *d* is mesh wire diameter or plate thickness, ${k}_{D}=\frac{{E}_{s}{d}^{3}(1-{A}_{s})}{12(1-{v}^{2})}$ is the mesh structure effective flexural stiffness, $E$ is the mesh material elastic modulus, $v$ is Poisson’s ratio and ${A}_{s}$ is open area aperture ratio. ${A}_{s}(\%)=\frac{a\times b}{{p}_{1}\times {p}_{2}}\times 100\%$, *p*_*1*_ and *p*_*2*_ are the gap of the centroid of two adjacent mesh wires.

$a$ (mm)	$b$ (mm)	$d$ (mm)	${A}_{s}$ (%)	${\theta }_{a}$	*k*_*D*_ (N/m)
–	–	0.18	0.00	156	0.06
0.45	0.45	0.25	61.17	155	0.11
0.75	0.75	0.40	62.24	153	0.44
0.86	0.86	0.42	64.54	150	0.48

2 $$\theta ={90}^{o}+arcsin \left (\frac{\Delta y}{R} \right)\mp {\alpha }_{BL}$$

where *∆y* is the vertical distance between the contact line and the fitted curve center, and *R* is the equivalent radius of the mathematical function fitted. The correction angle (${\alpha }_{BL}$) due to the inclination of the droplet contact line varies within 0° to 3°. The error estimated in the droplet contact angle measurement based on the several repeats is within 3%.

### Droplet dynamic behavior

**Figure 5:**
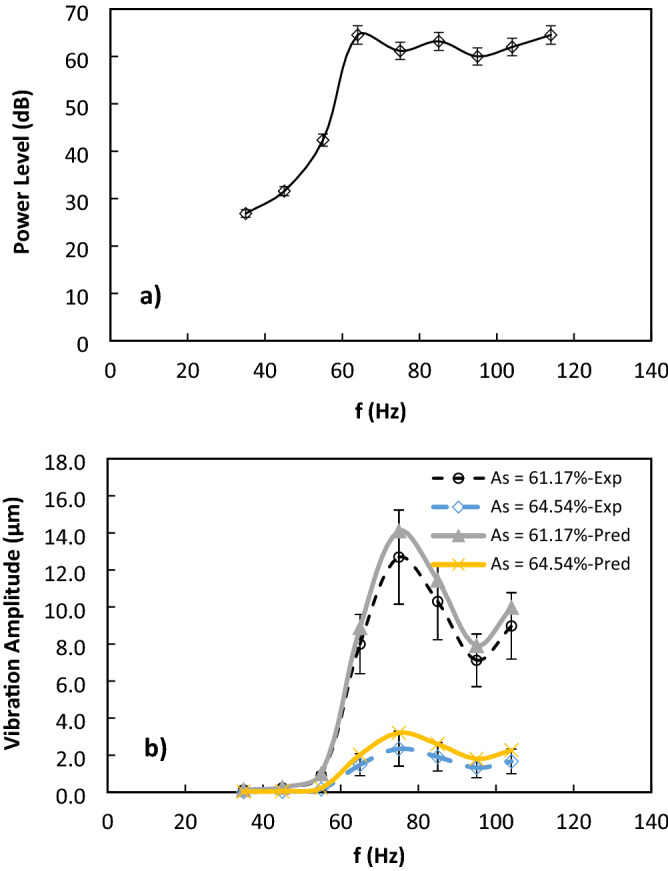
Variation of: (**a**) sonic power and (**b**) mesh screen displacement amplitude predicted and obtained from experiments with excitation frequency.

The mesh screen was sonically excited at 40 Hz to 120 Hz and 25 dB to 65 dB from the bottom of the mesh. The variation of sound power level with the excitation frequency is shown in Fig. 5a and the local maxima of power sound level occurs at 75 Hz sonic excitation frequency. The effective flexural stiffness (*k*_*D*_) of the mesh structure is given in Table 1. It is worth noting that the effective flexural stiffness is estimated from, ${k}_{D}=\frac{{E}_{s}{d}^{3}(1-{A}_{s})}{12(1-{v}^{2})}$ here, ${E}_{s}$ is the mesh material elastic modulus, $v$ is Poisson’s ratio and ${A}_{s}$ is open area aperture ratio. ${A}_{s}(\%)=\frac{a\times b}{{p}_{1}\times {p}_{2}}\times 100\%$, *p*_*1*_ and *p*_*2*_ are the gaps of the centroid of two adjacent mesh wires The effective flexural stiffness of the mesh screen increases with the open-cell aperture ratio due to increasing wire size diameter. Figure 5b depicts the vertical displacement at the center of the mesh surfaces for *A*_*s*_ = 61.17% and *A*_*s*_ = 64.54% and various excitation frequencies as measured with the aid of an accelerometer (KX132-1211 accelerometer, Kionix). The displacement is higher for mesh having a lower aperture ratio due to decreasing stiffness (Table 1). The mesh displacement is maximum for the sonic excitation frequency of 75 Hz corresponding to the natural frequency of the speaker diaphragm. The maximum vertical displacement of the vibrating mesh screen can also be predicted from the porous circular plate approach in which the droplet weight opposes the sound pressure force acting normal to the mesh surface^29^; hence resulting in the expression: ${\delta }_{{y}_{max}}=\frac{{p}_{rms}{R}_{s}^{2}}{64{k}_{D}}-\frac{{W}_{d}{R}_{s}^{2}}{16\pi {k}_{D}}$, here: ${p}_{rms}$ is sound pressure level, ${k}_{D}\sim \frac{{E}_{s}{d}^{3}(1-{A}_{s})}{12(1-{\nu }^{2})}$ is the effective stiffness of mesh screen, ${W}_{d}$ is the droplet weight, $d$ is wire diameter, $v$ is Poison ratio, ${R}_{s}$ is the radius of the top speaker opening and ${E}_{s}$ is the elastic modulus of steel. The vertical displacement amplitude is predicted using the expression for the various mesh/plate structures considered in the present study. Figure 5b shows that the predicted amplitudes are comparable to experimental measurements. Minor deviations observed can be due to the assumptions of applying the typical plate theory to the mesh structures, which exhibits interwoven circular rods array structures. Nevertheless, the prediction results follow the same trend as that of the experiment.

The external sonic excitation of the droplet results in the formation of capillary and gravity oscillations in the droplet fluid. Depending on the wavelength of the external oscillations, the capillary wave or the gravitational oscillations become dominant in the droplet fluid. Earlier, the natural oscillation of an inviscid droplet (*f*_*n*_) is related to^30^:

3 $${\left(\frac{{f}_{n}}{{f}_{c}}\right)}^{2}=\frac{1}{3\pi }n(n-1)(n+2)$$

where *f*_*c*_ (Hz) is the capillary frequency (${f}_{c}=\sqrt{\frac{\gamma }{m}}$, where *m* is the drop mass), *f*_*R*_ being the natural Rayleigh frequency and *n* is the mode of oscillation. As the external oscillation wavelength becomes shorter than the fluid capillary length $\sqrt{\frac{\gamma }{g\Delta \rho }}$, where *γ* is surface tension, *g* is gravity, and *Δρ* is the difference of fluid and air densities, then the effect of the capillary wave on droplet fluid oscillation becomes dominant^31^. Consequently, the natural frequency of the droplet oscillation can be deduced from the equation of motion (second-order linear homogenous ODE) in the form of^32^:

4 $${f}_{n}=\frac{{\omega }_{n}}{2\pi }=\sqrt{\frac{n\left(n-1\right)\left(n+2\right)\gamma }{4{\pi }^{2}\rho {R}^{3}}}$$

Here, ${\omega }_{n}$ is the natural frequency of oscillation (rad/s), *ρ* is the density of the droplet fluid and *R* is the droplet equivalent diameter. The natural frequency of the droplet oscillations varies depending on the modes (*n*) of oscillations. The natural frequencies predicted from Eq. (4) and measured for the droplet oscillation mode *n* = 3 are 67 Hz (prediction) and 63 ± 5 Hz (experiment) while the droplet oscillation mode of *n* = 4 is 101 Hz (prediction) and 98 ± 5 Hz (experiment). Moreover, the contact angle of the droplet changes during droplet fluid oscillations. The dependence of droplet contact angle on the oscillation frequency is formulated earlier^33^, and the condition satisfying the resonant oscillation of the droplet surface gives rise to the relations^34^: ${l}_{c}=2R\theta$ and ${l}_{c}=\frac{n\lambda }{2}$ or $\theta =\frac{n\lambda }{4R}$, here *l*_*c*_ is the circumferential length of the droplet over the surface, θ is the contact angle, R being the equivalent radius droplet curvature, λ is the oscillation wavelength. For the capillary waves, the frequency of the droplet surface oscillation (*f*_*c*_) takes the form^31^: ${f}_{c}=\sqrt{\frac{2\pi \gamma }{\rho {\lambda }^{3}}}$. Since the droplet with a small volume on the hydrophobic surface demonstrates an almost spherical cap shape, the droplet volume is associated with the contact angle and the drop mass (*m*). The resonant oscillation frequency, then, becomes^34^:

5 $${f}_{c}=\frac{\pi }{2}\sqrt{\frac{{n}^{3}\gamma }{24m}\frac{(co{s}^{3}\theta -3cos\theta +2)}{{\theta }^{3}}}$$

**Figure 6:**
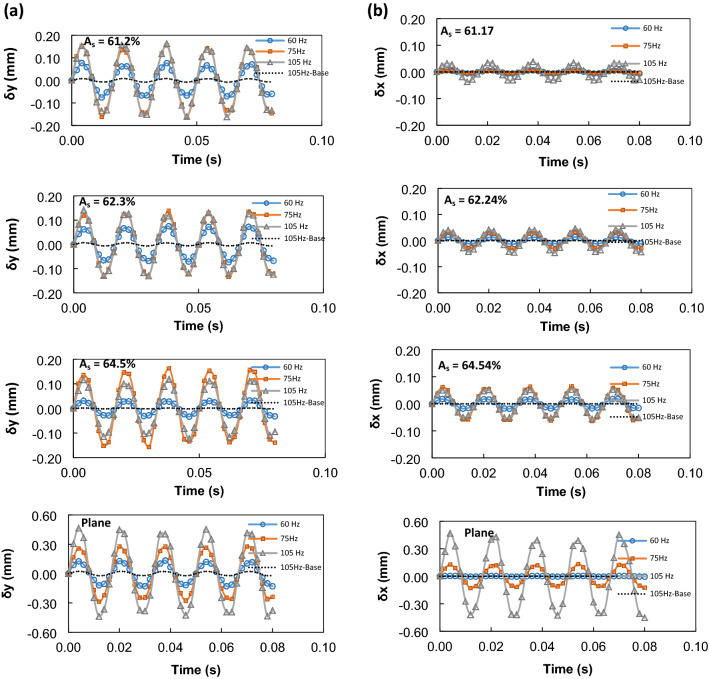
(**a**) Droplet maximum vertical displacement with time at different sonic excitations frequencies for different mesh screen open aperture ratio (*A*_*s*_) and plane hydrophobic surface. (**b**) Droplet maximum horizontal displacement with time at different sonic excitation frequencies for different mesh screen open aperture ratio (*A*_*s*_) and plane hydrophobic surface.

Equation (5) demonstrates that as the frequency of the oscillation changes, the contact angle of the droplet on the mesh surface changes. Nevertheless, the change of the contact angle for the external sonic excitation frequencies is small and as observed from the experiments, it is within the order of ± 3° at 105 Hz. The small variation of the contact angle with the frequency can be associated with the interfacial contact length over the mesh, which is greater than that of the droplet on the hydrophobic plain substrates. It is worth noting that the droplet on the mesh has interior and exterior contact lines because of the mesh screen open area. In addition, the pinning of the sessile droplet, which is associated with the surface tension at the interface, is related to $\sim \pi {D}_{c}\gamma cos{\theta }_{c}$, where *D*_*c*_ is the droplet contact length over the mesh, *θ*_*c*_ apparent contact angle. The gravitational and the pinning forces oppose the excitation force generated at the droplet bottom because of the pressure force created by the sonic excitation over the droplet exposed areas. Since the droplet apparent contact angle change is small, the effect of contact angle variation on the droplet oscillation frequency becomes small during the sonic excitations. This can be seen from Fig. 6a and b, in which the experimental data for the maximum vertical (Fig. 6a) and horizontal (Fig. 6b) displacement of the droplet surface are shown with time for different excitation frequencies and the mesh screen open aperture ratios (*A*_*s*_). The hydrophobic plain surface having the same droplet contact angle is also accommodated in Fig. 6 for comparison. The maximum displacement of the droplet surface represents the difference of displacement between the droplet under sonic excitations and without sonic excitation (sessile droplet) on the mesh surface. The vertical and horizontal displacements have almost the same peak values with time for all oscillation frequencies. Increasing frequency enhances the vertical and horizontal displacement of the droplet surface, i.e. droplet surface maximum height increases. The frequencies selected enable to keep the droplet over the mesh surface through the experiments, i.e., the droplet repelling because of detachment from the surface is avoided. Since the sonic excitation is applied from the droplet bottom side, the vertical displacement of the droplet surface is larger than the horizontal displacement. Droplet oscillation creates wobbling of the droplet over the mesh surface and the frequencies incorporated does not result in the droplet rolling or sliding motions over the surface. Moreover, the influence of the mesh screen open aperture ratio (*A*_*s*_) on the maximum vertical displacement is not significant (Fig. 6a); hence, the maximum vertical displacement slightly decreases with increasing the mesh screen open aperture ratio. This is attributed to the balance of bending forces acting on the exciting mesh structures, i.e., mainly due to the droplet weight and sound pressure. However, a small increase in the horizontal displacements is observed (Fig. 6b) such that increasing the mesh screen open aperture ratio enhances the vertical displacement of the droplet. This becomes more apparent for the mesh screen open aperture ratio of *A*_*s*_ = 64.5%. The predictions show that the vibrational amplitude of the mesh screen decreases with increasing mesh open area owing to the decrease in mesh screen stiffness. This indicates that the extended contact region of the droplet bottom surface results in the larger vertical wobbling. The plain hydrophobic surface results in a larger contact surface than the meshes; hence, the three-phase contact line remains smaller than that of the mesh because of the droplet fluid interface with the wire surfaces. This lowers the pinning force of the droplet over the surface. The sonic excitations, therefore, can create a larger vertical and horizontal displacement of the droplet surface, which can be observed when comparing the horizontal and vertical displacements of the droplet at various frequencies. Hence, the droplet stability under large wobbling over the sonic excitations becomes questionable for the plain hydrophobic surface. The experimental observations demonstrate that the droplet tends to roll over the hydrophobized plain surface as the excitation frequency increases beyond 105 Hz. The forced vibration of a liquid droplet on a vibrating hydrophobic surface can be expressed with the non-linear equation for the single-degree-of-freedom system based on the Duffing equation^8,35^, i.e., ${m}_{d}\ddot{\delta }+\eta \dot{\delta }+k\delta +\beta {\delta }^{3}=Fcos(\omega t+\varphi )$, here: $\delta$ is vertical droplet displacement, ${m}_{d}$ is droplet mass, $\eta$ is viscosity, $k={C}_{1}\gamma$ is stiffness which is related to the surface tension, $F={m}_{s}{\delta }_{{y}_{max}}4{\pi }^{2}{f}_{f}^{2}$ is the droplet excitation force term, ${f}_{f}$ is forcing frequency of exciter (Hz), ${m}_{s}$ is mass of mesh screen, $\beta$ is a constant, and $\varphi$ is the phase angle. The solution of the single-degree-of-freedom non-linear system leads to the non-linear expression for the amplitude of droplet lobe vertical displacement^35^, i.e., $\delta \cdot \sqrt{{\left({4{\pi }^{2}(f}_{0}^{2}-{f}_{f}^{2})+\frac{3}{4}\beta {\delta }^{2}\right)}^{2}+\frac{{4\eta }^{2}{\pi }^{2}{f}_{f}^{2}}{{m}_{d}^{2}}}={m}_{s}{\delta }_{{y}_{max}}4{\pi }^{2}{f}_{f}^{2}$. For mesh structure with an open aperture ratio of *A*_*s*_ = 61.17% (i.e., ${f}_{0}=63 Hz$, ${f}_{f}=60 Hz$, ${\delta }_{{y}_{max}}=8.87 \mu m$, $\eta =1\times {10}^{-3} Pa s$, ${m}_{s}=24\times {10}^{-3} kg$, $\beta =-3 x {10}^{-10}$ and ${m}_{d}=8\times {10}^{-6}$ kg), the amplitude of vertical droplet displacement becomes 0.052 mm, which reasonably compares well to the 0.097 mm predicted from the experiments.

**Figure 7:**
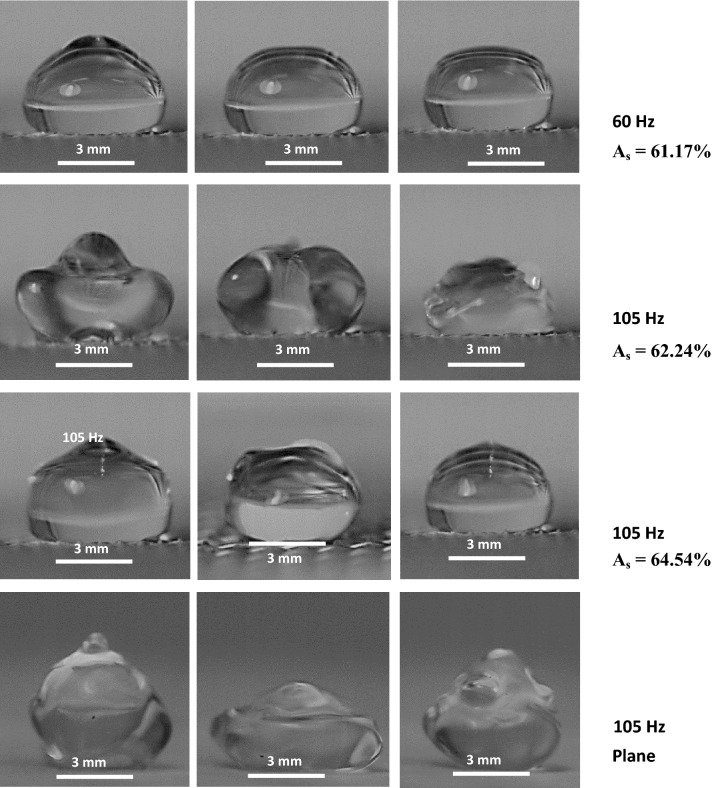
Optical images of droplets obtained from high-speed camera for various mesh screen open aperture ratio (*A*_*s*_) and plane hydrophobic surface.

Figure 7 shows the optical views of the droplet obtained from the high-speed recording at various excitation frequencies at three different times. The vertical displacement of the droplet and mode of oscillation change as the frequency increases. However, the distinct behavior of the droplet can be observed for mesh and plain hydrophobic surfaces. The maximum droplet surface vertical displacement remains higher for the case of plane hydrophobic surface than that of the droplet on the mesh surface. On the other hand, the dispersion relation of the hydroelastic waves in the droplet fluid located on the hydrophobic surface plays an important role in categorizing the waves in terms of their wavelengths. In general, the dispersion relation of the hydroelastic waves in the liquid over the surface of a plate takes the form^36^:

6 $${f}^{2}=\left(gk+\frac{\gamma }{\rho }{k}^{3}+\frac{{k}_{D} }{\rho }{k}^{5}\right)tanh(kh)$$

Here, *f* is the frequency, *k* is the wavenumber, *k*_*D*_ is the effective flexural stiffness, and *h* is the liquid depth. The dominant fluid force can define the hydroelastic wave types. In this case, the first term on the right side of Eq. (6) resembles gravity, the second term is the surface force, and the third term is bending force; hence, the gravity and surface tension forces generally define the wave dynamics in the fluid as the drop is located on the stationary and stiff surface^37^. The Capillary waves are the results of the interaction due to the surface and inertial forces. The gravity waves are generated as the gravitational force [first term in Eq. (6)] dominates over the surface tension and bending forces [second and third terms in Eq. (6)]. However, the hydroelastic forces can also be grouped in line with their wavelengths. In this case, three main scales could be introduced based on the force ratios. These are^38^:

7 $${\lambda }_{g\gamma }=2\pi \sqrt{\frac{\gamma }{\rho g}} : {\lambda }_{\gamma {k}_{D}}=2\pi \sqrt{\frac{{k}_{D}}{\gamma }} : {\lambda }_{g{k}_{D}}=2\pi \sqrt{\frac{{k}_{D}}{\rho g}}$$

here, ${k}_{D}$ is the effective flexural stiffness of the plate, ${\lambda }_{g\gamma }$ is the gravity-capillary wavelength, ${\lambda }_{\gamma {k}_{D}}$ is the flexural-capillary wavelength, and ${\lambda }_{g{k}_{D}}$ is the flexural-gravity wavelength. As the wavelength becomes longer the influence of the forces creating the wave becomes dominant in the fluid^37^. After knowing that the mesh vertical and horizontal displacements are small as compared to droplet fluid during the oscillations, the oscillation wavelengths, which are greater than the gravity-capillary wavelength (${\lambda }_{g\gamma }$), governs the droplet oscillatory behavior during the excitation. Alternatively, for the droplet located on the hydrophobic mesh surfaces, the resonant modes can occur on the surface and the nodal patterns are created on the droplet geometry, which can be observed from the optical images (Fig. 7). For the gravity-capillary dominant waves in the droplet, the resonant frequency (*f*_*res*_) can be arranged to yield^39^:

8 $${f}_{res}\approx \sqrt{\frac{2\pi {n}^{3}\gamma }{\rho {l}_{c}^{3}}}$$

where ${l}_{c} = 2\pi R$, *R* being the droplet radius and *n* is the integer number and takes the values 2, 3, 4, and more The first resonance frequency measured affecting the droplet shape is at about 67 Hz while its counterpart predicted from Eq. (8) is about 67. 8 Hz for *n* = 3. However, the small differences in the measured and predicted [Eq. (8)] resonant frequencies are associated with the assumption made in Eq. (8), which is $n\left(n-1\right)\left(n+2\right)\approx {n}^{3}$. Nevertheless, the difference in prediction and measurement for the resonant frequency is small.

**Figure 8:**
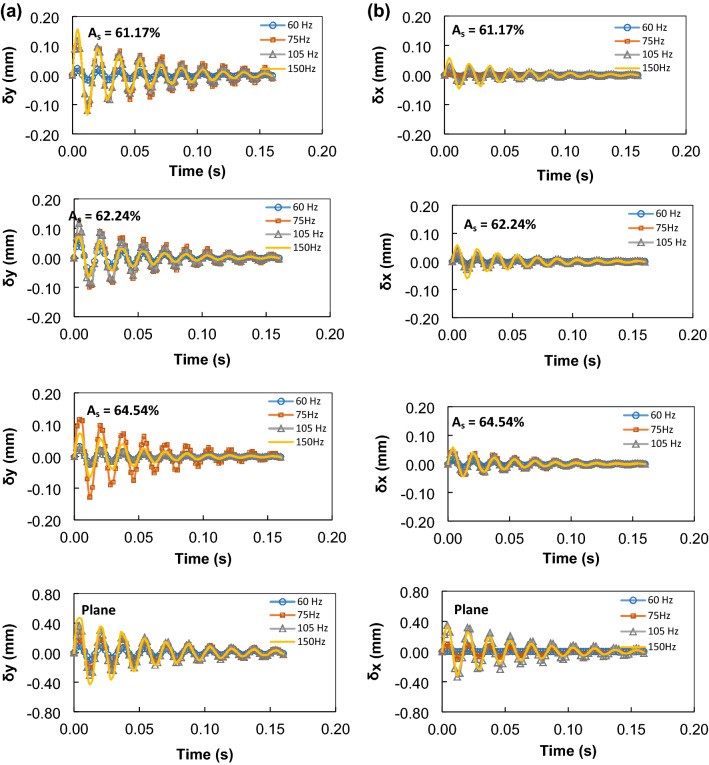
(**a**) Maximum vertical displacement of the droplet with time at different sonic excitation frequencies for different mesh screen open aperture ratio (*A*_*s*_) and plane hydrophobic surface after shot-off sonic excitation. (**b**) Maximum horizontal displacement of the droplet with time at different sonic excitation frequencies for different mesh screen open aperture ratio (*A*_*s*_) and plane hydrophobic surface after shot-off sonic excitation.

Figure 8a and b show the decay of the droplet maximum displacement along the x, and y-axis for various sonic excitation frequencies and different mesh screen open aperture ratios (A_s_). It is worth mentioning that the displacement is the difference between the maximum and minimum droplet vertical top height after the ending of the sonic excitations. In this case, the droplet maximum displacement is related to the oscillations after the sonic excitations are shot off. The contact angle variation during droplet oscillation can affect the damping of the maximum displacement with time after the excitation is shot-off^40^. However, this influence is found to be small due to the large contact line on the hydrophobic mesh surface, which is similar to the bubbles on the hydrophobic surface. Hence, the droplet pinning force becomes larger while reducing the droplet contact angle variation with the excitation frequencies. Therefore, the dependence of the contact angle variation on the damping becomes weak and the peaks of the maximum displacement occur at the same time during shot-off period. Moreover, the decay rate of the maximum displacement amplitude provides the damping rate of the droplet oscillations. The decay rate of the droplet amplitude follows ${e}^{-\delta t}$, where δ is the damping factor. The damping factor reduces as the mesh screen open aperture ratio reduces. For the droplet excitation at 105 Hz and, after the shot-off, the damping factor for the vertical oscillation changes for the plain and mesh screen open aperture ratios, i.e. the damping factor takes the values of 15.6 s^−1^, 17.5 s^−1^, 19.04 s^−1^, and 34.08 s^−1^ for 61.2%, 62,3%, and 64.5% of mesh screen open aperture ratios, and the plain surface, respectively. However, the damping factor differs for the horizontal oscillation of the droplet, i.e. it takes the values 3.27 s^−1^, 5.12 s^−1^, 9.76 s^−1^, and 19.54 s^−1^, for the plain and mesh screen open aperture ratios of 61.2%, 62,3%, and 64.5%, respectively. The damping factor remains larger along with the vertical oscillation of the droplet than that of the horizontal motion. In addition, the damping factor for the plain hydrophobic surface remains lower than that corresponding to the droplet motion on the mesh surface for all excitation frequencies. Consequently, the mesh surface allows relatively gradual decay of the droplet oscillatory motion than that occurring on the plaint surface. In general, the damping in the droplet occurs because of the viscous dissipation and surface tension effects. The influence of the viscosity and surface tension on the damping can be estimated, in relative terms, from the ratio of viscous to surface tension forces^4^. This yields the expression $\sim \frac{\mu f{D}^{2}}{\gamma }$, where μ is droplet fluid viscosity, *D* is the droplet equivalent diameter, *f* is in the order of 2πω. In this case, 60 μL water droplet leads the force ratio in the order of 9 × 10^–6^, which is considerably small. Hence, the influence of the viscous dissipation on the droplet damping is negligibly small. In addition, the bulk viscous damping factor can be associated with the fluid viscosity, the wavenumber, and the density in the form of^40^: $\frac{2\mu }{\rho }{k}^{2}$, where k is the wavenumber $k=\frac{2\pi }{\lambda }$, here λ is the wavelength of the oscillation. The damping factor at 60 Hz of the droplet oscillation yields 0.14 s^−1^, which is much smaller than that estimated from the experiments for 60 μL droplet on a mesh screen open aperture ratio of *A*_*s*_ = 61.2, which is (7.8 s^−1^). The surface tension waves, which are created during the droplet oscillation, disturb the flow streaming and can cause streamline turbulence^41^. This, in turn, can cause turbulent kinetic energy dissipation and can contribute to the damping in the droplet oscillation. Since the excitation frequencies are low (60 Hz to 105 Hz range), the kinetic energy dissipation is considered to be small^39^ and its contribution to the damping is expected to be very small. However, surface tension waves create small-sized spiky oscillations on the droplet surface, which is observed during the experiments.

## Conclusion

The behavior of a water droplet on a hydrophobic mesh and subjected to the sonic excitation from the droplet bottom is considered and the influence of the mesh screen open area on the droplet oscillation is examined. The metallic meshes having square screen open areas are incorporated and the mesh surface is hydrophobized by depositing functionalized nanosize silica particles. The hydrophobized surface results in the contact angle of 150°–155° ± 3° and hysteresis of 4° ± 2°. Increasing the mesh screen open area changes the droplet contact angle; however, the hysteresis remains almost the same. A high-speed recording system and a tracker program are used to quantify the natural and resonant frequencies of the droplet oscillations. Increasing sonic excitation frequency enhances the droplet maximum displacement in both horizontal and vertical plains, which is more pronounced for large mesh screen open aperture ratios (*A*_*s*_); however, the vertical displacement remains larger than the horizontal displacement of the droplet. In addition, for a plain hydrophobic surface with the same contact angle, the vertical and horizontal displacements become larger than that corresponding to the mesh. Hence, droplet oscillation remains critical for droplet mobility over the plain hydrophobic surface, i.e. droplet can be rolled off over the hydrophobic plain surface for the frequency ranges at which the droplet remains sessile on the mesh surface. The first resonance frequency measured affecting the droplet shape is at about 67 Hz while its counterpart predicted is about 67. 8 Hz, which occurs for *n* = 3. The damping factor for the droplet oscillation along the vertical and horizontal plains change; in which case, the damping factor attains larger values for the vertical plane than the horizontal plane. In addition, increasing sonic excitation frequency enhances the damping factor of the droplet. The surface tension waves create small-sized spiky oscillations on the droplet surface, which are observed from the high-speed data as recorded during the experiments.
